# High Resolution Ultrasound and Photoacoustic Imaging of Orthotopic Lung Cancer in Mice: New Perspectives for Onco-Pharmacology

**DOI:** 10.1371/journal.pone.0153532

**Published:** 2016-04-12

**Authors:** Florian Raes, Julien Sobilo, Marilyne Le Mée, Stéphanie Rétif, Sharuja Natkunarajah, Stéphanie Lerondel, Alain Le Pape

**Affiliations:** 1 PHENOMIN-TAAM-UPS44, CIPA (Centre d’Imagerie du Petit Animal), CNRS Orléans, France; 2 INSERM U1100, CEPR, University of Tours, France; French National Centre for Scientific Research, FRANCE

## Abstract

**Objectives:**

We have developed a relevant preclinical model associated with a specific imaging protocol dedicated to onco-pharmacology studies in mice.

**Materials and Methods:**

We optimized both the animal model and an ultrasound imaging procedure to follow up longitudinally the lung tumor growth in mice. Moreover we proposed to measure by photoacoustic imaging the intratumoral hypoxia, which is a crucial parameter responsible for resistance to therapies. Finally, we compared ultrasound data to x-ray micro computed tomography and volumetric measurements to validate the relevance of this approach on the NCI-H460 human orthotopic lung tumor.

**Results:**

This study demonstrates the ability of ultrasound imaging to detect and monitor the *in vivo* orthotopic lung tumor growth by high resolution ultrasound imaging. This approach enabled us to characterize key biological parameters such as oxygenation, perfusion status and vascularization of tumors.

**Conclusion:**

Such an experimental approach has never been reported previously and it would provide a nonradiative tool for assessment of anticancer therapeutic efficacy in mice. Considering the absence of ultrasound propagation through the lung parenchyma, this strategy requires the implantation of tumors strictly located in the superficial posterior part of the lung.

## 1. Introduction

Because lung cancer still remains the leading cause of cancer-related death, there is a need to develop more accurate and predictive preclinical protocols and relevant cancer models. Orthotopic lung cancer models have the advantage of being more predictive regarding clinical relevance, including the ability of primary tumors to develop spontaneous metastasis but also more predictive regarding the therapeutic response. The implementation and exploration of such orthotopic models allows us to improve our understanding of the biology of cancer to interpret preclinical *in vivo* results in humans, especially for the potential therapeutic response of anticancer agents. Studies taking into account more representative parameters from clinical situations, particularly hypoxia, are of great interest to boost innovation for new anticancer treatments [1–3].

One important parameter in oncology is tumor volume assessment before but also during treatments [4]. In a clinical setting, the pulmonary tumor measurements are predominantly performed with X-ray computed tomography (CT) imaging [5]. For pulmonary preclinical oncology, imaging objectives are to improve the accuracy for determining volumes, without irradiation effects or interferences with the anti-tumor response.

Thanks to technological developments for both X-ray sources and detectors, CT dedicated to small animal imaging provides a sub-millimetric resolution making this tool efficient for the characterization of lung tumor volumes. However, the radiation dose delivered to tumors remains a limitation, especially when a study requires repeated exams [6].

Bioluminescence imaging (BLI) brought about a revolution in preclinical oncology research but this method provides quantitative information about tumor proliferation without any possible sizing. Moreover, since BLI is dependent upon metabolism, it is not reliable when tumors become hypoxic [7].

In clinical practices, lung ultrasound (US) has been gaining in popularity among clinicians and has become an essential tool in critically ill management [8,9]. However regarding human pulmonary oncology, there is no possible use of US except for invasive endoscopy of cancer nodules and lymph nodes [10,11]. The main limitation of endoscopy and ultrasound is the detection of these nodules if proximity with the probe is not close enough. This access limitation is due to the absence of US propagation through the lung parenchyma because of air.

On the contrary, preclinical high resolution US and photoacoustic imaging (PAI) are promising modalities to investigate lung tumor progression and hypoxia respectively but considering the specific constraints of US, the implantation of tumors in the superficial posterior lung region is required.

The large cell NCI-H460 orthotopic lung carcinoma model that we chose to improve, is based on a study by Gagnadoux *et al*. [12], leading to the growth of a solitary intrapulmonary nodule located near the posterior diaphragmatic surface.

Here we recommend refining such an onco-pharmacology protocol in a translational approach while overcoming physical US limitations allowing lung tumor exploration. In this longitudinal study we assessed orthotopic lung tumor volumes in mice by *in vivo* 3D US and also hypoxic tumor status by PAI. Furthermore, we compared our data to different imaging methods with the aim to validate this new approach.

## 2. Materials and Methods

### 2.1 Ethics Statement

All procedures on animals were performed in accordance with European ethical guidelines (European directives 2010/63/EU) and were approved by the Regional Committee for Animal Care and Ethics in Animal Experiments (C2EA-03 Comité d’éthique en expérimentation animale Campus CNRS d’Orléans).

### 2.2 Cell Culture

The NCI-H460-luc2 human lung cancer cell line was obtained from Perkin Elmer (France). This cancer cell line was maintained according to the supplier’s instructions.

### 2.3 Animals

Pathogen-free 6 to 8 week-old female nude Balb/c mice were purchased from Charles River Laboratories (France). Mice were acclimated for 7 days in the laboratory before experimentation and were maintained in sterilized filter-stopped cages inside a controlled ventilated rack (USA) with access to food and water *ad libitum*. They were examined daily for clinical signs, distress, decreased physical activity and weighed 3 times a week.

### 2.4 Subcutaneous and Intra-Bronchial Cell Xenograft

Human lung cancer xenografts from NCI-H460-luc2 cells were established in Balb/c nude mice. We first performed subcutaneous implantation in order to get reference data from measurement techniques on standard conditions, so that 10 mice were anaesthetized by inhalation of 1.5% isoflurane with air (Isoflo^®^, AXIENCE S.A.S, France) and inoculated by different tumor burdens (either 1x10^5^ to 2.5x10^6^ tumor cells in 100 μL PBS) in the dorsal flank. For orthotopic implantation, 24 mice were inoculated (1.25x10^5^ or 2.5x10^5^ tumor cells in 25 μL PBS) using a 1.9F×50cm blunt-end silicon catheter inserted into the bronchus via a laryngoscope (S1A Fig). This delicate procedure to get superficial cell deposition into a posterior part of a lower lobe via a main bronchus requires interventional imaging. The position of the radio-opaque catheter is checked by planar radiography (Faxitron MX20, Faxitron X-ray corp, USA) (S1B Fig) then actual deposition of 99mTc-labelled tumor cells is controlled by Single Photon Emission Computed Tomography (Nano Spect CT, Mediso, Hungary), (S1C Fig).

### 2.5 Computed Tomography

Mice were anesthetized by 1.5% isoflurane and placed on a bed in prone position. For tumor measurements, computed tomography was performed using a small animal imager (eXplore CT 120, Trifoil Imaging, USA) with an external respiratory gating device (Biovet, USA). Some mice received an intravenous injection of the vascular contrast agent eXIA160^™^ (Binitio Biomedical, Canada). Tumor volumes were obtained by manually delineating margins of tumors from sagittal sections of CT images using Microview analysis+ 2.3 software.

### 2.6 Bioluminescence Imaging

BLI was performed once a week until the end of the study (Day 28) using an IVIS-Lumina II (Perkin Elmer, France) generating a pseudo-colored image representing light intensity and superimposed over a greyscale reference image. Each mouse was IP injected with 100mg/kg luciferin potassium salt (Promega, France). Mice anesthetized by 1.5% isoflurane were placed on a thermostatically controlled heating pad (37°C) during imaging. Acquisition binning and duration were set depending on tumor activity. Signal intensity was quantified as the total flux (photons/seconds) within ROIs drawn manually around the tumor area using Living Image 4.0 software (Perkin Elmer, France). The sum of signals from the prone and supine positions was considered for each mouse.

### 2.7 Ultrasound and Photoacoustic Imaging

Mice anesthetized by 1.5% isoflurane were placed on a thermostatically controlled heating pad in prone position with the paws taped over the ECG electrodes attached to the table. Respiratory gating was derived from ECG. A colorless aqueous warmed ultrasonic gel (Supragel^®^, LCH, France) without any air bubbles was applied between the skin and the transducer. Tumors were imaged with the VisualSonics Vevo^®^LAZR System (FUJIFILM VisualSonics Inc, Canada). 3D scans of US image being recorded digitally. The tumor area in coronal planes was measured by manually delineating margins using Vevo^®^LAB 1.7.2 software. The software then calculated the corresponding volume of each coronal slice. For hypoxia assessments, tumors were investigated by PAI with OxyHemo-Mode so that average values of SO_2_ were determined and corresponding hypoxic volumes documented. Tumor perfusion status and VEGFR2 expression were assessed by contrast enhanced ultrasound (CEUS) imaging following IV injection (tail vein) of Vevo MicroMarker^™^ and Target-Ready MicroMarker^™^ coupled with either anti-VEGFR2 or isotype control antibodies (eBioscience, USA). Imaging protocols were performed with the destruction-replenishment sequences. Data were processed with the VevoCQ^™^ software. A key parameter that should be respected for accurate 3D US acquisition is the positioning of the transducer (S2A Fig). The US beam has to be directed towards the lung tumor with the best angle possible, so that the entire tumor can be detected during the acquisition. Despite the presence of artifacts and shadows due to the ribs, when changing the angle of the transducer it is possible to select the most efficient positioning for the transducer that allows detection. The minimal size of tumors that can be detected is 1mm in diameter, however the bigger the tumor size, the fewer artifacts from the ribs impact tumor detection. Transducers with central frequency at 21MHz and 40MHz, were used for B-Mode imaging of large and small tumors respectively. PAI was performed with the 21MHz transducer.

### 2.8 Sacrifice and Organ Removal

Mice under anesthesia were sacrificed by cervical dislocation and tumors were collected from each animal for immediate *ex vivo* assessments. In order to assess the accuracy of *in vivo* US measurements, tumors were collected at the end of the study (Day 28) and were imaged *ex vivo* with 3D US. The dedicated plate filled with ultrasonic gel allowed us to measure tumor volumes accurately, avoiding any motion of collected tissues due to the movement of the US transducer during the 3D acquisition (S2B Fig).

### 2.9 Volumetric Measurements

Tumors were carefully dissected then immersed in suitable graduated cylinders filled with water. Volumes were assessed by measuring the weight of water removed from the cylinder to make the concave meniscus adjusted to the upper edge of the baseline graduation mark (S2C Fig). These measurements were performed in triplicate.

### 2.10 Statistical Analysis

Statistical analysis was performed using GraphPad Prism software version 5.0 (GraphPad, USA). Correlation graphs and R squared coefficients were obtained by nonlinear regression.

## 3. Results

### 3.1 Validation of Techniques for Tumor Volume Measurements on Subcutaneous Models

**Fig 1:**
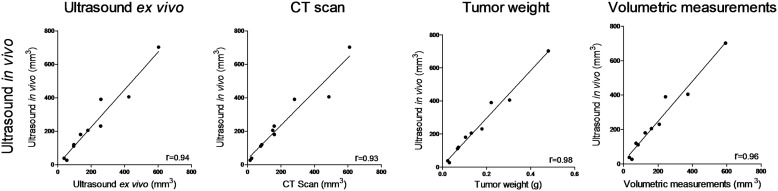
Correlation analysis between different methods for tumor volume assessment. Nonlinear regression of data points collected from orthotopic subcutaneous tumors (n = 10 animals). The correlation coefficient R squared is provided in the lower right hand of each graph.

To assess the intrinsic accuracy of each modality to determine tumor volumes, the subcutaneous tumor model allows us to avoid disrupting contributions such as respiratory movements and well-known problems associated with US chest exams. Comparison of results was achieved from subcutaneous tumors with sizes ranging from 15 to 700mm^3^. Correlation analysis between US measurements achieved *in vivo* Vs *ex vivo* (R^2^ = 0.94), Vs CT (R^2^ = 0.93), or tumor weight (R^2^ = 0.98) and volumetric assessments (R^2^ = 0.96) clearly validated the ability of US and CT protocols to achieve accurate determination of tumor volumes (Fig 1).

### 3.2 Control of the Tumor Cell Implantation

For subcutaneous tumors, all the 10 xenografted animals were enrolled for assessment of the tumor sizes when determined by the various measurement methods. For orthotopic lung tumors, seven days after engraftment the tumor growth was confirmed by BLI (S1D Fig), either in the left or right lung. Based on BLI intensity, 12 mice with tumor activity ranging from 7.45x10^5^ to 8.55x10^7^ Photons/sec were selected for the study.

### 3.3 Assessment of Tumor Volumes in Lung by *In Vivo* US Imaging

**Fig 2:**
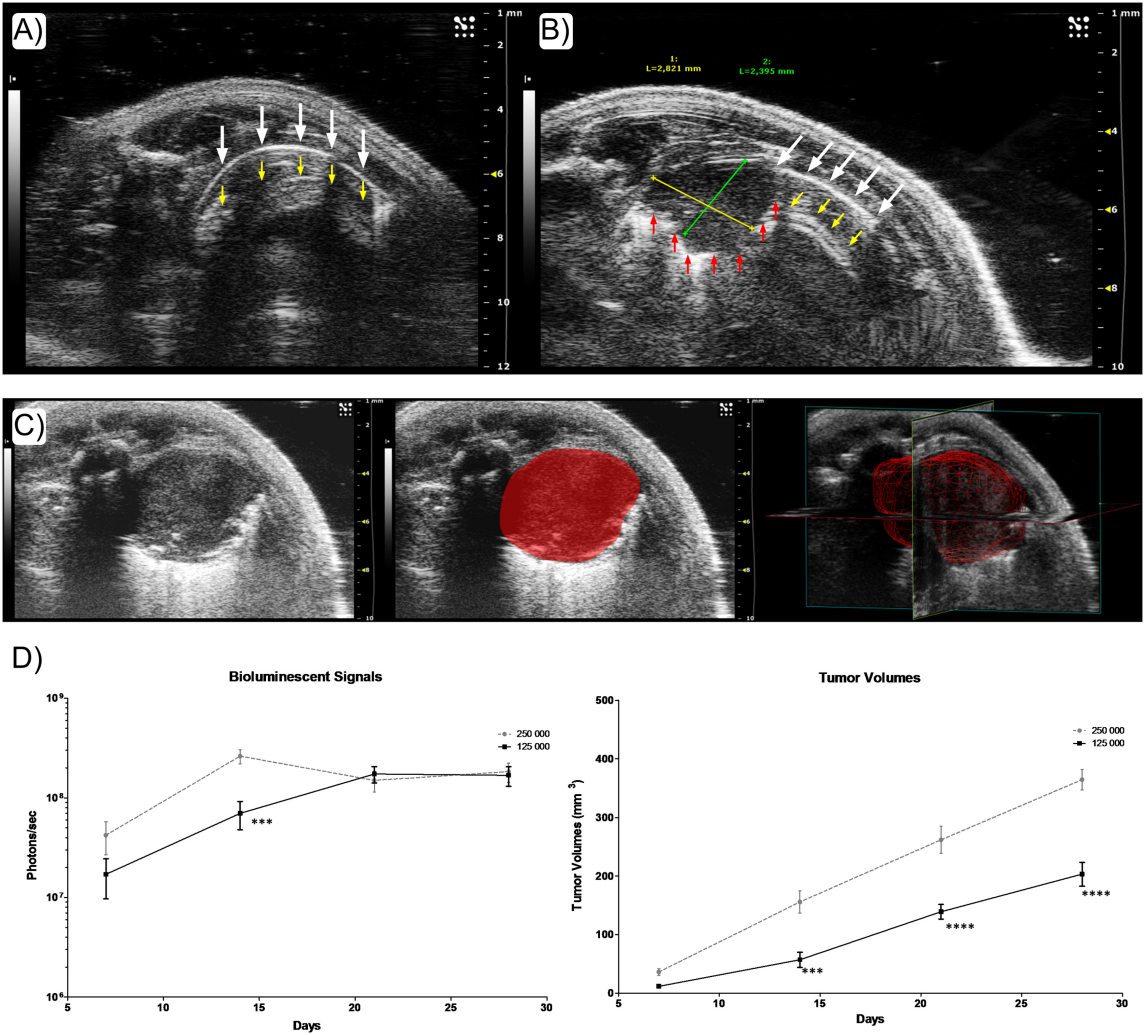
*In vivo* identification and monitoring of orthotopic lung tumors. (A) & (B) Detection of lung tumors by High Resolution Ultrasound Imaging. (A) 2D B-Mode acquisition on a healthy lung in mouse. Vertical white arrows point out the pleural line. Vertical yellow arrows correspond to A lines, representing reverberations of the pleural line. (B) On 2D B-Mode Ultrasound imaging of a lung bearing an orthotopic NCI-H460luc tumor (2.8mmx2.4mm), vertical red arrows point out the margins of the tumor, highlighted by the typical bright shadow artifact. We also remark white and yellow arrows indicating the pleural line and A lines respectively. (C) From 2D to 3D Ultrasound B-Mode imaging of a lung tumor in mouse. The red area corresponds to the lung tumor in the thoracic cavity of the mouse. The red grid corresponds to the tumor volume obtained by tracing margins on each 2D B-mode slices from the 3D acquisition. (D) Assessing tumor burden with BLI (left) and US (right), data are presented as mean ±SEM and statistically analyzed. A two-way repeated-measure analysis of variance followed by Bonferroni post-tests was used for the data of over time course. Differences were considered significant at p< 0.05. Left: Signal intensity from *in vivo* longitudinal monitoring of tumor proliferation by BLI following the deposition of 1.5x10^5^ or 2.5x10^5^ tumor cells (Photons/sec). Right: *In vivo* tumor volumes measured by US imaging using a transducer mounted on a 3D motor, comparing the tumor growth between 2 different tumor burdens (mm^3^). Results represent mean±SEM (n = 5 animals per groups). (***p<0.001; ****p<0.0001).

As in human lung US, we observed artifacts from the pleural line due its echogenicity. As pointed out by the white arrows (Fig 2A), the pleural line appears bright, as it is an increased ultrasound reflection at the interface between pleura and healthy lung. We noticed motionless regularly spaced hyperechogenic lines, named A lines, which are repetition artifacts of the pleural line. The break in the pleural and A lines allowed us to identify the lung tumor with certainty, even at early stages (Fig 2B). A typical artifact observed below the lung tumor, such as a bright shadow corresponding to the meeting point between tumor and healthy pulmonary parenchyma, enabled us to identify tumor margins. This posterior enhancement is explained by the difference in ultrasound velocity between tumor and lung parenchyma.

Positioning the transducer with an appropriate angle made it possible to record successive 2D images with a stepper motor for 3D acquisitions. Processed volumes were displayed as a red grid (Fig 2C).

When compared to US tumor volumes in the two different groups of lung tumors, BLI exhibited very different patterns of evolution (Fig 2D). In the first group (1.25x10^5^cells), BLI increased progressively until day 21 then remained unchanged whereas US determined volumes increased during the entire study. In the second group (2.5x10^5^cells), constant increase in volumes determined by US was quite different from the BLI pattern exhibiting pronounced regression from day 14 to day 21, then stagnation. *In vivo* US follow up of lung tumors allowed us to measure tumor volumes precisely and highlighted the significant differences between growth curves from the two initial tumor burdens.

### 3.4 Comparison of Techniques for Tumor Volume Measurements in Lungs

**Fig 3:**
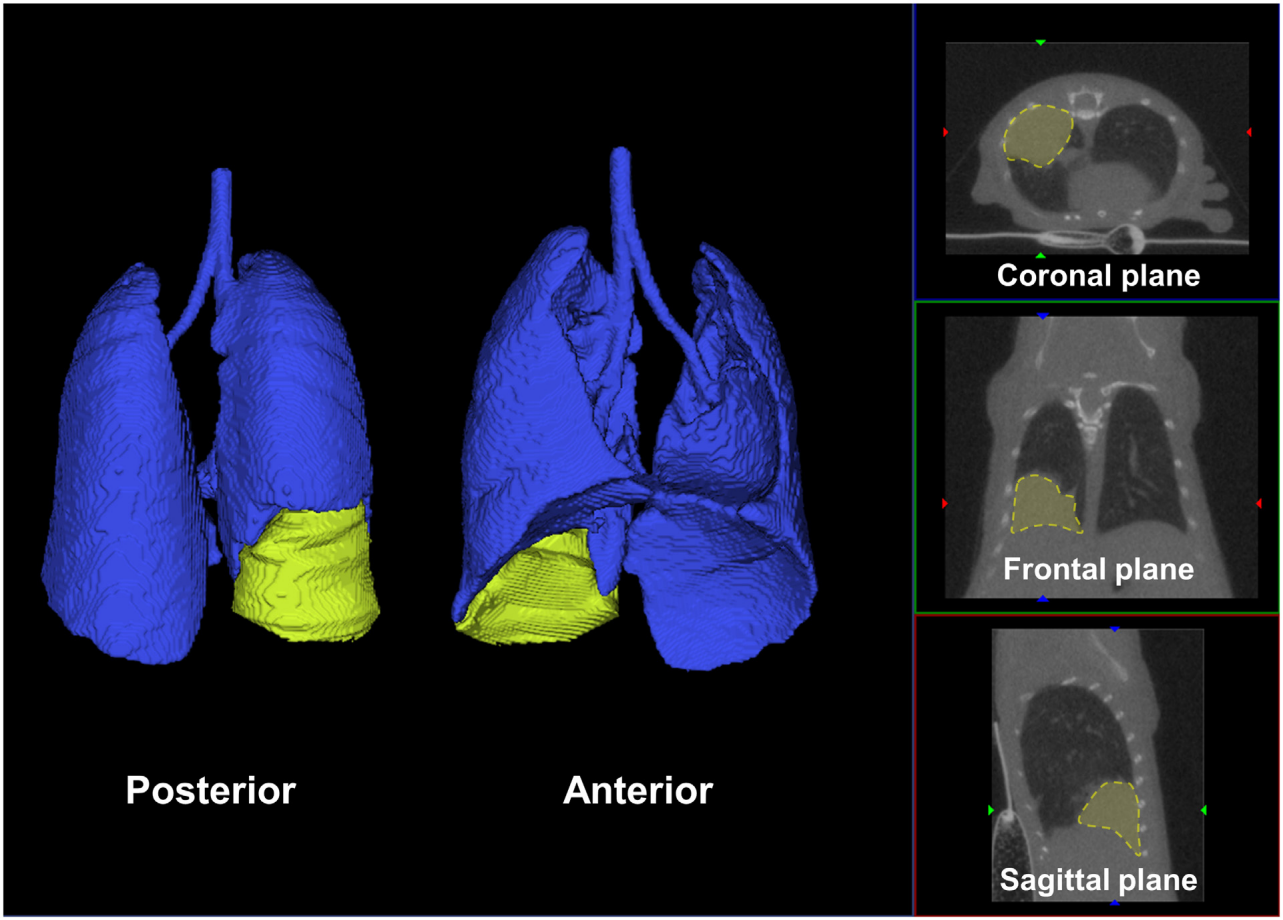
Assessment of tumor volumes with micro Computed Tomography. 3D rendering of CT scan anterior and posterior reconstructions, after processing the tumor delineation on 2D slices from coronal, frontal or sagittal plane. Healthy lung parenchyma is blue whereas tumor is highlighted in yellow.

At the end of the study (Day 28), non-contrast enhanced CT was performed with the aim to compare data from US and CT. The 3D reconstituted volumes of healthy lungs and tumors were processed (Fig 3). Isosurfaces of healthy lungs were obtained with an automated segmentation whereas tumor volumes required manual delineation on 2D slices.

**Fig 4:**
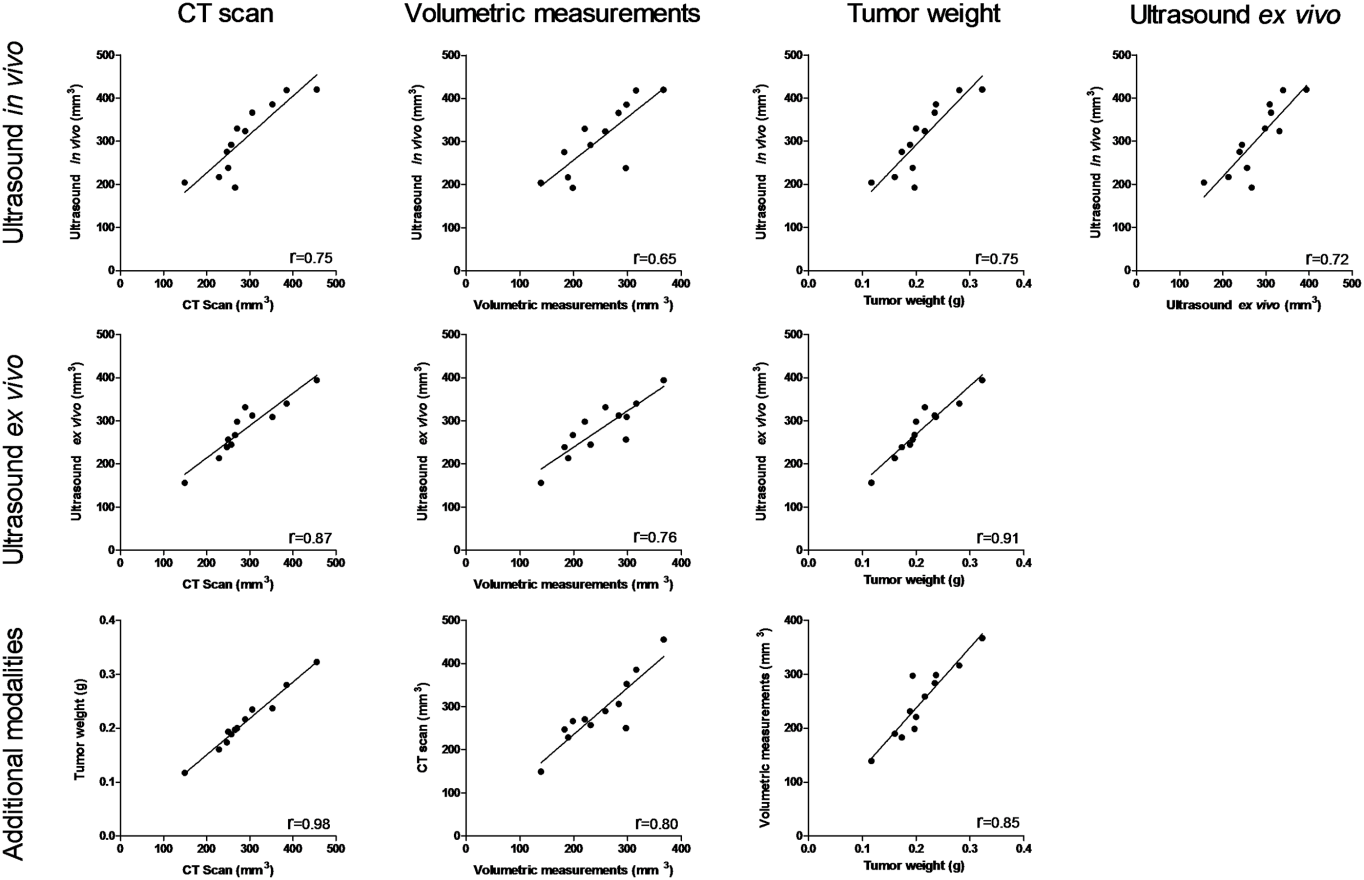
Correlation analysis between different modalities for tumor volume assessment. Nonlinear regression of data points collected from orthotopic lung tumors (n = 12 animals). These graphs compare the correlation between 3D US imaging *in vivo* and *ex vivo*, *in vivo* 3D CT scans, *ex vivo* volumetric measurements and tumor weight.

The correlation analysis of data obtained by each method on orthotopic lung tumors is reported in Fig 4. We compared tumors with different sizes, data obtained from *in vivo* and *ex vivo* US, CT, volumetric measurements and weights. CT scan provides an accurate estimation of tumor volumes compared to *ex vivo* US (R^2^ = 0.87), tumor weight (R^2^ = 0.98) and volumetric measurements (R^2^ = 0.80).

For *in vivo* measurements, results clearly indicate gross correlation of volumes processed with *in vivo* US, CT and tumor weight (R^2^ = 0.75). These results do not reflect an overestimation of tumor volumes measured by US or CT as compared to the tumor weight. Such a correlation is also observed with ex vivo US and volumetric measurements (R^2^ = 0.72 and R^2^ = 0.65 respectively).

### 3.5 Contrast Enhanced Ultrasound, Power Doppler and Photoacoustic Imaging

**Fig 5:**
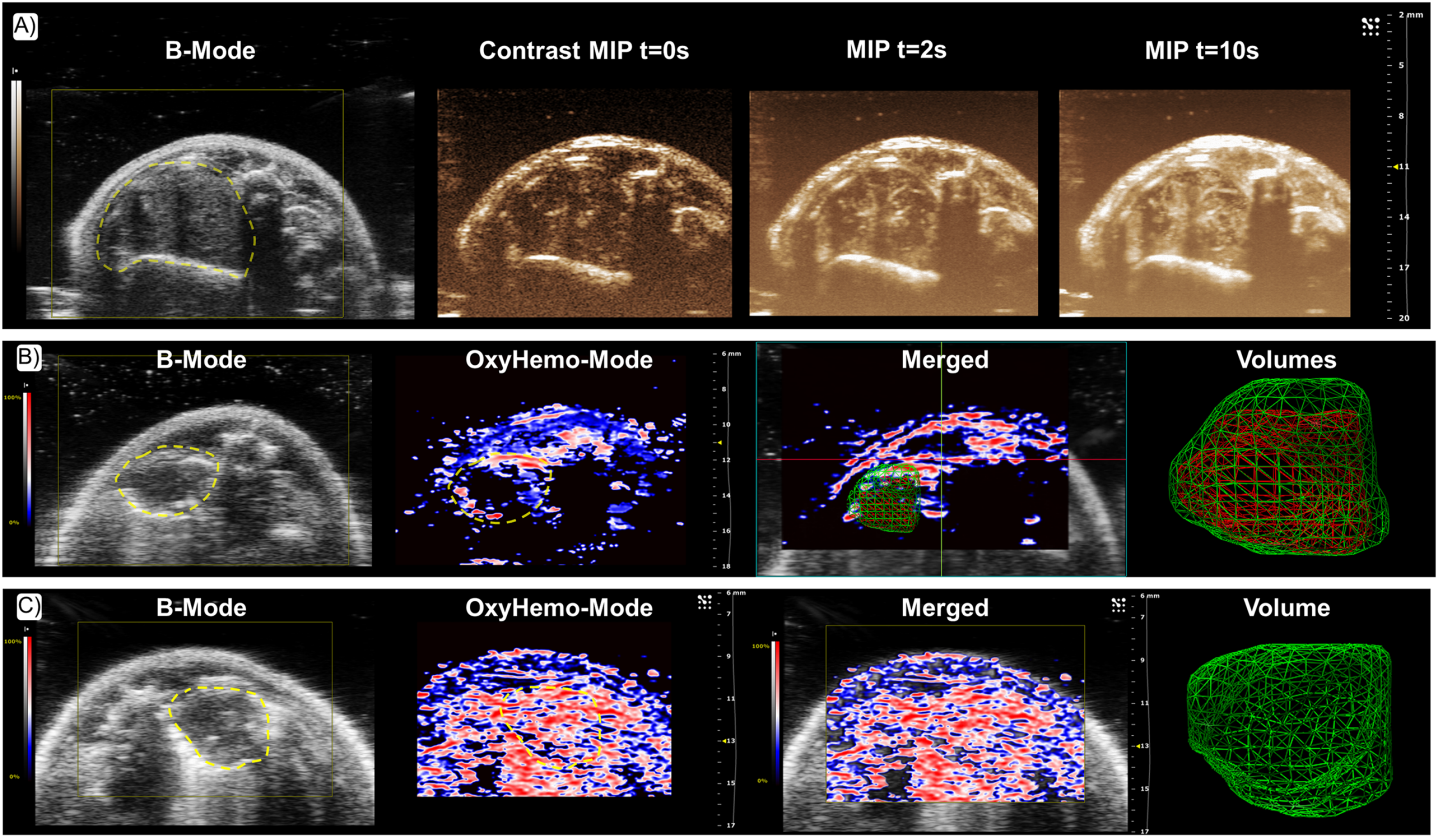
Contrast enhanced ultrasound imaging and Photoacoustic imaging of hypoxia on orthotopic lung tumors in mice. (A) B-mode image of a lung tumor with corresponding contrast image before IV injection of Vevo MicroMarker^™^. Maximum Intensity Projection after injection of MicroMarker^™^. (B) B-mode image of a hypoxic lung tumor with corresponding OxyHemo photoacoustic images. With the OxyHemo-Mode, red areas indicate well oxygenated parts of the tumor whereas blue and dark areas indicate the presence of hypoxia. Regarding the 3D volumes, the red grid corresponds to the hypoxic region of tumor and green grid corresponds to the entire tumor. (C) B-mode image of a well oxygenated lung tumor with corresponding OxyHemo photoacoustic images showing absence of any hypoxic core.

Since it was possible to perform *in vivo* US B-Mode imaging, the ability to measure different US and PAI parameters was investigated. Interestingly we noticed that micro US imaging enabled the visualization of tumor perfusion status after the injection of MicroMarker^™^ contrast agent (Fig 5A). We noticed a well enhanced signal through contrasted enhancement demonstrating an efficient perfusion status. CEUS imaging was more efficient and sensitive in highlighting vascularization parameters than Power Doppler. Thanks to Power Doppler (no injection of contrast agent required), we were able to observe the larger vessels. Due to the tiny size of other vessels and contribution of parameters such as slow blood flow motion, heartbeats and breathing, the visualization of small blood flows is challenging, even with ECG and breathing gating (data not shown).

Regarding PAI, we obtained information on the oxygenation status of tumors and it was possible to reveal the presence of hypoxia inside tumors noninvasively (Fig 5B). We noticed oxygenated areas in the periphery of the tumor as compared to the hypoxic core. 3D acquisitions allowed quantification of hypoxia and volumes for both regions (V_tumor_ = 29.4mm^3^, SO2_tumor_ = 11.4%; V_hypoxia_ = 10.2mm^3^, SO2_hypoxia_ = 0.4%, all numbers given as single values for the tumor shown here) (Fig 5B). It is important to observe that some mice with comparable total tumor volumes exhibited a well oxygenated tumor (V_tumor_ = 47.3mm^3^, SO2_tumor_ = 51.7%, all numbers given as single values for the tumor shown here) (Fig 5C).

**Fig 6:**
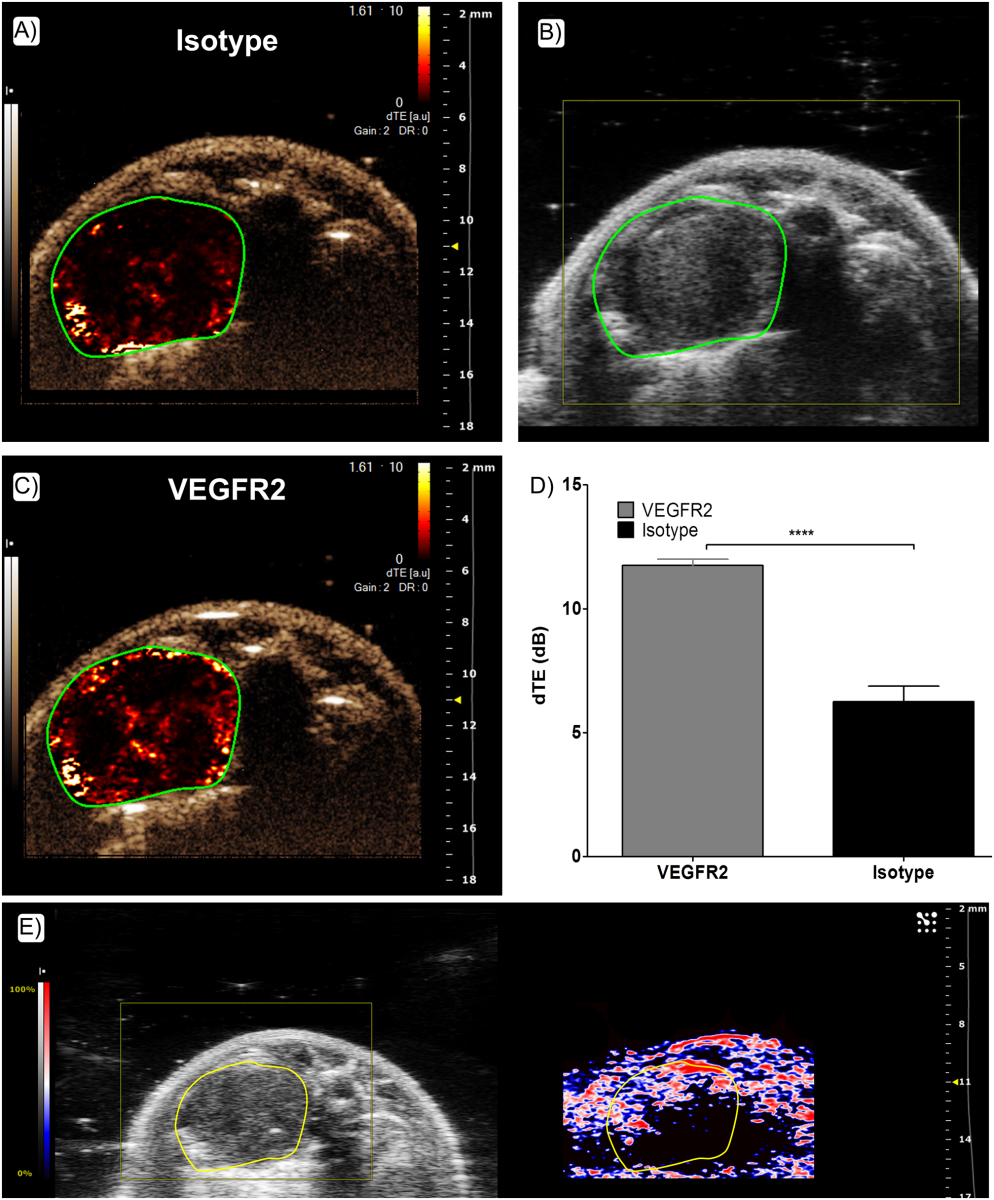
Molecular imaging of VEGFR2 by Targeted Contrast Enhanced Ultrasound Imaging. (A) & (C) Parametric images of the spatial distribution of contrast agent bubbles. (A) Isotype control conjugated microbubbles. (C) VEGFR2 conjugated microbubbles (Target Ready Vevo MicroMarker^™^). (B) Corresponding B-Mode image of the tumor. (D) Differential Targeted enhancement of VEGFR2 and Isotype control conjugated microbubbles (dTE corresponds to the difference between the echo power from both targeted and free bubbles, and the echo power from free bubbles only). Statistical analysis was performed with the Student's unpaired t test (n = 4 animals per group). (****p<0.001). (E) Corresponding PA image highlighting hypoxic areas where VEGFR2 is mainly expressed.

Targeted CEUS allowed us to assess the relative VEGFR2 expression in lung tumors. The VevoCQ software calculated the differential Targeted Enhancement (dTE) values for each region of interest, the same analysis was completed for both the VEGFR2 and isotype control antibody conjugated contrast agents. From parametric images the spatial distribution of the bound contrast agent can clearly be seen (Fig 6A and 6C). Comparison of dTE between microbubbles labeled with VEGFR2 and isotype control antibodies was significant (Fig 6D). Moreover, hypoxic areas determined by PAI matched the spatial distribution of VEGFR2 (Fig 6E).

### 3.6 Limitations of Imaging Modalities

**Fig 7:**
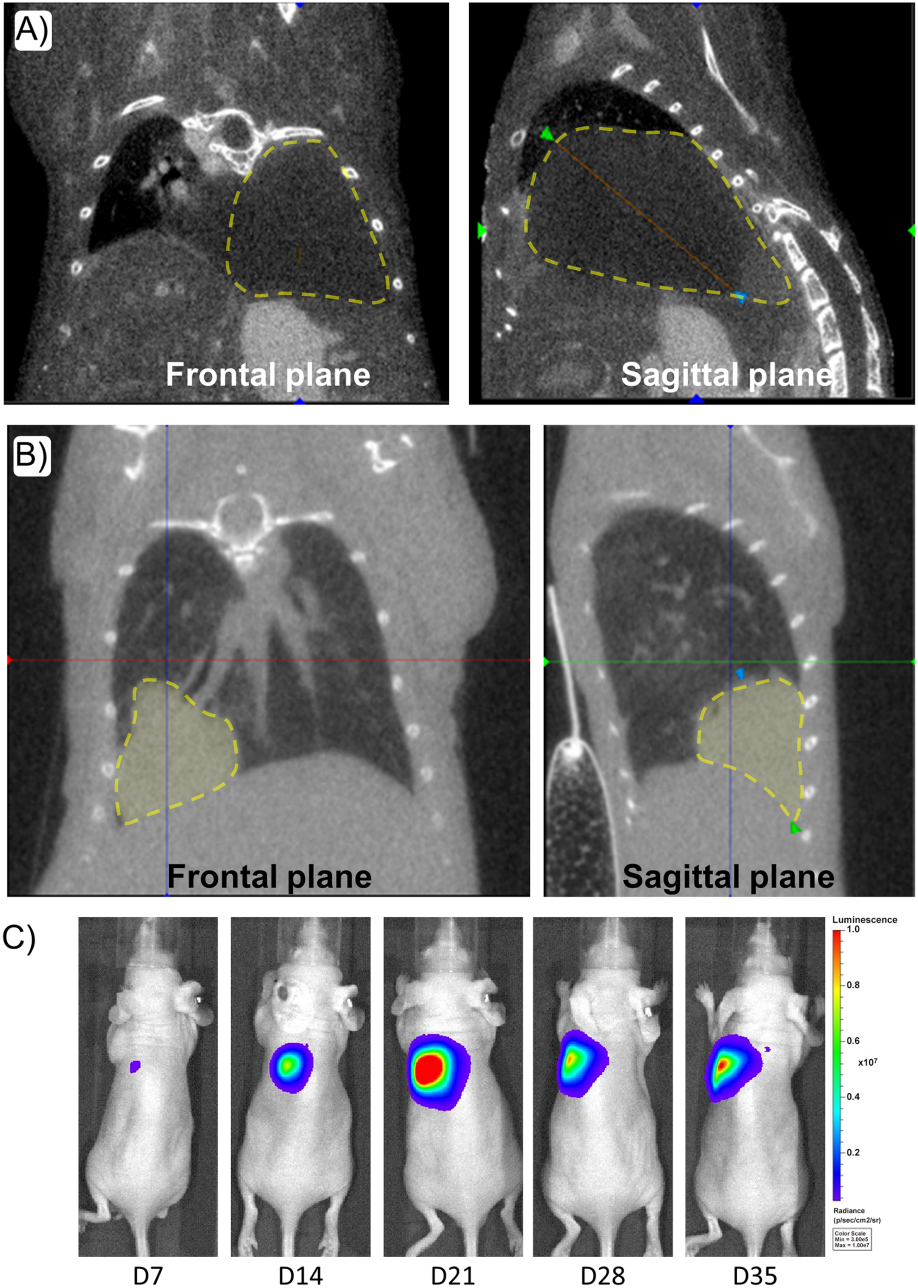
CT scans and bioluminescence signals of mice bearing orthotopic lung tumors. (A) 2D CT scan of a mouse bearing a lung tumor, after IV injection with 100μL of a mix with PBS and eXIA 160, a iodinated vascular contrast agent. Delineation of the tumor is visible on both planes with a yellow dashed line. (B) CT scan obtained without contrast agent injection. Delineation of the tumor is visible on both planes with a yellow dashed line. (C) Longitudinal BLI study on a mouse bearing a lung tumor between day 7 and day 35 (Photons/sec/cm^2^/steradian), after implantation of 1.25x10^5^ tumor cells.

CT images performed following vascular contrast agent injection allowed clear delineation of the tumor from surrounding tissues (more specifically the liver) (Fig 7A). On the contrary, non-contrast enhanced CT performed on this orthotopic lung cancer model was more difficult to analyze especially for large tumors, resulting in less accurate determination of tumor volume (Fig 7B).

Fig 7C corresponds to the evolution of BLI signals within the same animal over time. These results demonstrate that BLI signals from the lung tumor were disrupted during the tumor growth since an increase in BLI was observed up to day 21 then regression was observed whereas the tumor volume was still increasing (Fig 2D).

## 4. Discussion

The use of preclinical tumor models allows us to follow biological parameters or tumor growth and test the efficacy of therapeutic agents. The experiments described here have proven their utility in providing researchers a new competitive technique to assess orthotopic lung tumor growth by US imaging *in vivo*, allowing non-invasive and non-radiation based investigations.

Each of the imaging modalities used during this study has advantages and disadvantages. CT is relevant to image the respiratory system owing to the low density within the lung space [13]. Regarding lung cancer detection, CT has been proven to be useful in detecting lung tumors located in the apex and hilar area but less efficient for tumors located near the diaphragmatic surface [14], because the lack of contrast with the underlying tissue makes it difficult to measure larger tumor volumes and requires extensive knowledge of both CT imaging and contrast agents with animal anatomy. The use of CT vascular contrast agents, such as eXIA^™^, was necessary to perform accurate measurements of tumor volume even if tumors were small and growing around the periphery of the lungs. Unfortunately, repeated use of contrast agent is not recommended in onco-pharmacological studies due to potential effects on tumor growth or therapies. The main limitation of CT imaging is the radiation dose delivered to the tumor. Indeed in this radiosensitive tumor model, the impact of repeated imaging examinations (1 per week versus 1 per 2 weeks, with a conventional chest CT scan delivering 38.9±3.9 Gy) on tumor growth is significant. The influence of doses on tumor proliferation and strong risk of potentiation of an anti-tumor effect may affect the relevance of data about efficacy assessment of anticancer agents [6,15,16].

Although BLI is a quantitative technique exploited routinely to assess tumor proliferation, it is strictly dependent upon metabolic conditions, namely the presence of ATP and O_2_. As shown in Fig 7C, the initial tumor burden in these mice was small (1.25x10^5^ cells), nevertheless leading to the formation of a hypoxic core and to the decrease of the bioluminescent signal. These tumor hypoxic conditions are a critical point to be considered to ensure the relevance of obtained data [7,17,18]. Because both BLI and US are non-invasive, measurements were performed longitudinally in the same animal. Results confirmed that quantification of BLI signals from the lung is delicate, and obtained data should be used with caution regarding the tumor growth. Nevertheless BLI still remains a great resource in the early stage of studies to control induction of lung tumors and for allocation of animals into homogenous groups.

Among other available imaging modalities, US is noninvasive, non-radiating, does not necessarily require contrast agents and thus offers the advantage of being fast, cheap and with minimal impact on the investigated process. Moreover, this method is not subject to disruption due to the appearance of hypoxia. Given that the shape of tumors is not ellipsoid 3D acquisitions were necessary to accurately measure volumes. Combined with high resolution US imaging, PAI is a real-time noninvasive and quantitative imaging modality for the study of tumor hypoxia and heterogeneity. SO_2_ mapping can be performed thanks to the existing differences in optical absorption between oxygenated and deoxygenated hemoglobin [19]. PAI, combining high optical absorption contrast with ultrasound high resolution, provides both anatomical and functional data. In oncology, numerous parameters have to be assessed on tumors, therefore this multimodality is quite relevant to monitor therapeutic response or disease burden. However, due to the limitation of US propagation through the lung parenchyma, the translation to clinical investigations cannot be considered.

Thanks to ultrasound molecular imaging, it was possible to assess the expression and spatial distribution of VEGFR2-targeted microbubbles as compared with microbubbles labeled with isotype control antibodies. Differences in acoustical echoes between free bubbles and those linked to the molecular target are the key feature to facilitate discrimination [20]. This opens up interesting perspectives to characterize changes at a molecular level [21] and for investigations on targeted therapies [22], more especially anti-angiogenic strategies based on anti-VEGFR treatments [23].

In conclusion, we described for the first time a Photoacoustic and Ultrasound imaging strategy to investigate orthotopic lung tumor growth and assess key biomarkers such as hypoxia or VEGFR2 expression *in vivo*. Hypoxia is a major parameter involved in lung tumor resistance towards radio and chemotherapies. For personalized medicine, hypoxia can be assessed by PET tracers labelled with ^18^Fluorine [24,25] and chemotherapies dedicated to hypoxic tumors have already been included in phase III clinical trials [26,27]. This medium/high throughput validated imaging resource would be of great interest to longitudinally follow both tumor growth and hypoxic status in animals when testing the efficacy of new anticancer therapies. This avoids any risk of disruption of tumor progression compared to other imaging methods such as BLI and CT.

Considering its ability to provide high resolution molecular imaging, it is feasible to imagine an additional potent application of PAI for intra-tumoral micro-biodistribution of therapeutic agents such as monoclonal antibodies [28].

## Supporting Information

S1 Fig Orthotopic lung tumor implantation and assessment of the engraftment. (A) The diagram represents the route of inoculation. The catheter is inserted in the deep bronchus through the trachea, so that the tumor grows in the lower lobes of the lungs, near the posterior diaphragm surface. (B) The in vivo control of the catheter positioning by planar X-ray is performed in order to avoid the implantation in the wrong site. (C1) Control of the accuracy of cell deposition into the deep bronchus by SPECT/CT imaging of 99Tcm-labeled cells. (C2) Sagittal view demonstrating the suitable location, at the posterior part of the lung. (D) Tumor growth is confirmed by BLI on day 7.
(TIF)

S2 Fig In vivo and ex vivo measurement methods to determine the lung tumor volumes. (1) Transducer positioning allowing for the conduction of ultrasound through the tumor parenchyma (delineated yellow area). (2) Set up for ex vivo 3D US acquisitions. An excavated plate is filled with ultrasound conductive gel around the tumor tissue in order to avoid any movement, and the transducer is positioned above. (3) Volumetric determination by immersing the tumor in a graduated cylinder filled with water. The water is removed from the graduated cylinder to adjust the concave meniscus at the upper edge of the baseline graduation mark and then weighed.
(TIF)

## References

[1] Walsh JC, Lebedev A, Aten E, Madsen K, Marciano L, Kolb HC. The clinical importance of assessing tumor hypoxia: relationship of tumor hypoxia to prognosis and therapeutic opportunities. Antioxid Redox Signal 2014;21(10): 1516–1554. 10.1089/ars.2013.5378 24512032 PMC4159937

[2] Graves E, Vilalta M, Cecic IK, Erler JT, Tran PT, Felsher D, et al Hypoxia in models of lung cancer: Implications for targeted therapeutics. Clin Cancer Res 2010;16(19): 4843–4852. 10.1158/1078-0432.CCR-10-1206 20858837 PMC2948600

[3] Le QT, Chen E, Salim A, Cao H, Kong CS, Whyte R et al An evaluation of tumor oxygenation and gene expression in patients with early stage non-small cell lung cancers. Clin Cancer Res 2006;12(5):1507–1514. 16533775 10.1158/1078-0432.CCR-05-2049

[4] Foster FS, Hossack J, Adamson SL. Micro-ultrasound for preclinical imaging. Interface Focus 2011;1(4): 576–601. 10.1098/rsfs.2011.0037 22866232 PMC3262267

[5] Fushiki H, Miyoshi S, Noda A, Murakami Y, Sasaki H, Jitsuoka M, et al Pre-clinical validation of orthotopically-implanted pulmonary tumor by imaging ^18^F-fluorothymidine-positron emission tomography/computed tomography. Anticancer Research 2013;33:4741–4750. 24222108

[6] Andouard S, Guilhem MT, Le Rouzic G, Sobilo J, Le Pape A, Lerondel S. Impact of radiation dose delivred by repeated CTscan examinations on tumor growth in preclinical model of lung cancer. Personal Communication 2011.

[7] Khalil AA, Jameson MJ, Broaddus WC, Lin PC, Dever SM, Golding SE, et al The influence of hypoxia and pH on bioluminescence imaging of luciferase-transfected tumor cells and xenografts. Int J Mol Imaging 2013;287697 10.1155/2013/287697 23936647 PMC3723249

[8] Lichtenstein DA. Lung ultrasound in the critically ill. Ann Intensive Care 2014;4:1 10.1186/2110-5820-4-1 24401163 PMC3895677

[9] Gargani L. Lung ultrasound: a new tool for the cardiologist. Cardiovasc Ultrasound 2011;9:6 10.1186/1476-7120-9-6 21352576 PMC3059291

[10] Wallace MB, Pascual JMS, Raimondo M, Woodward T, McComb BL, Crook JE, et al Minimally invasive endoscopic staging of suspected lung cancer. J Am Med Assoc 2008;299(5):540–546. 10.1001/jama.299.5.540 18252884

[11] Colella C, Vilmann P, Konge L, Clementsen PF. Endoscopic ultrasound in the diagnosis and staging of lung cancer. Endosc Ultrasound 2014;3(4):205–212. 10.4103/2303-9027.144510 25485267 PMC4247527

[12] Gagnadoux F, Le Pape A, Lemarié E, Lerondel S, Valo I, Leblond V, et al Aerosol delivery of chemotherapy in an orthotopic model of lung cancer. Eur Respir J 2005;26:657–6. 16204597 10.1183/09031936.05.00017305

[13] Wathen CA, Foje N, Avermaete T, Miramontes B, Chapaman SE, Sasser TA, et al In vivo X-ray computed tomographic imaging of soft tissue with native, intravenous, or oral contrast. Sensors 2013;13(6): 6957–6980. 10.3390/s130606957 23711461 PMC3715264

[14] Paulus MJ, Gleason SS, Kennel SJ, Hunsicker PR, Johnson DK. High resolution X-ray computed tomography: an emerging tool for small animal cancer research. Neoplasia 2000;2,62–70. 10933069 10.1038/sj.neo.7900069 PMC1531867

[15] Willekens I, Buls N, Lahoutte T, Baeyens L, Vanhove C, Caveliers V, et al Evaluation of the radiation dose in micro-CT with optimization of the scan protocol. Contrast Media Mol Imaging 2010;(4):201–7. 10.1002/cmmi.394 20665903

[16] Kagadis GC, Loudos G, Katsanos K, Langer SG, Nikiforidis GC. In vivo small animal imaging: Current status and future prospects. Med Phys 2010;37(12):6421–6442. 21302799 10.1118/1.3515456

[17] Lerondel S, Le Pape A. Bioluminescence imaging in rodents: When light illuminates research. Curr Mol Imaging 2013;2,18–25.

[18] Brulle L, Vandamme M, Ries D, Martel E, Robert E, Lerondel S, et al Effects of a non thermal plasma treatment alone or in combination with gemcitabine in a MIA PaCa2-luc orthotopic pancreatic carcinoma model. PLoS One 2012;7(12): e52653 10.1371/journal.pone.0052653 23300736 PMC3530450

[19] Needles A, Heinmiller A, Ephrat P, Bilan-Tracey C, Trujillo A, Theodoropoulos C, et al Development of a combined photoacoustic micro-ultrasound system for estimating blood oxygenation. IEEE Int Ultrason Symp Proc 2010;390–393.

[20] Rooij T, Daeichin V, Skachkov I, De Jong N, Kooiman K. Targeted ultrasound contrast agents for ultrasound molecular imaging and therapy. Int J Hyperthermia 2015;31(2):90–106. 10.3109/02656736.2014.997809 25707815

[21] Kaneko OF, Willmann JK. Ultrasound for molecular imaging and therapy in cancer. Quant Imaging Med Surg 2012;2(2):87–97. 23061039 10.3978/j.issn.2223-4292.2012.06.06 PMC3466813

[22] Hyvelin JM, Tardy I, Arbogast C, Costa MC, Emmel P, Helbert A, et al Use of ultrasound contrast agents microbubbles in preclinical research. Invest Radiol 2013;48(8):570–83. 10.1097/RLI.0b013e318289f854 23511194

[23] jiang Y, Allen D, Kerseman, Devery A M, Bokobza S M, Smart Sean, et al Acute vascular response to cediranib treatment in human non-small-cell lung cancer xenografts with different tumour stromal architecture. Lung cancer 2015;90:191–198. 10.1016/j.lungcan.2015.08.009 26323213 PMC4641245

[24] Borad M, Reddy S, Bahary N, Uronis H, Sigal D, Cohn A, et al Randomized phase ii trial of gemcitabine plus th-302 versus gemcitabine in patients with advanced pancreatic cancer. J Clin Oncol 2014; 33(13):1475–81. 10.1200/JCO.2014.55.7504 25512461 PMC4881365

[25] Chawla S, Cranmer L, Van Tine B, Reed D, Okuno S, Butrynski J, et al Phase II Study of the safety and antitumor activity of the hypoxia-activated prodrug th-302 in combination with doxorubicin in patients with advanced soft tissue sarcoma. J Clin Oncol 2014; 32(29):3299–306. 10.1200/JCO.2013.54.3660 25185097 PMC4588714

[26] Zegers C, van Elmpt W, Reymen B, Even A, Troost E, Ollers M, et al In vivo quantification of hypoxic and metabolic status of nsclc tumors using [^18^F]HX4 and [^18^F]FDG-PET/CT imaging. Clin Cancer Res 2014; 20(24):6389–97. 10.1158/1078-0432.CCR-14-1524 25316821 PMC5228298

[27] Peeters S, Zegers C, Biemans R, Lieuwes N, van Stiphout R, Yaromina A, et al TH-302 in combination with radiotherapy enhances the therapeutic outcome and is associated with pretreatment [^18^F]HX4 hypoxia pet imaging. Clin Cancer Res 2015;21(13):2984–92. 10.1158/1078-0432.CCR-15-0018 25805800

[28] Maillet A, Guilleminault L, Lemarié E, Lerondel S, Azzopardi N, Montharu J, et al The airways, a novel route for delivering monoclonal antibodies to treat lung tumors. Pharm Res 2011; 28:2147–2156. 10.1007/s11095-011-0442-5 21491145

