# Supplementary figures and images for: High Resolution Ultrasound and Photoacoustic Imaging of Orthotopic Lung Cancer in Mice: New Perspectives for Onco-Pharmacology
Source: PLoS One. 2016 Apr 12;11(4):e0153532. doi: 10.1371/journal.pone.0153532 (PMC4829195; doi:10.1371/journal.pone.0153532)

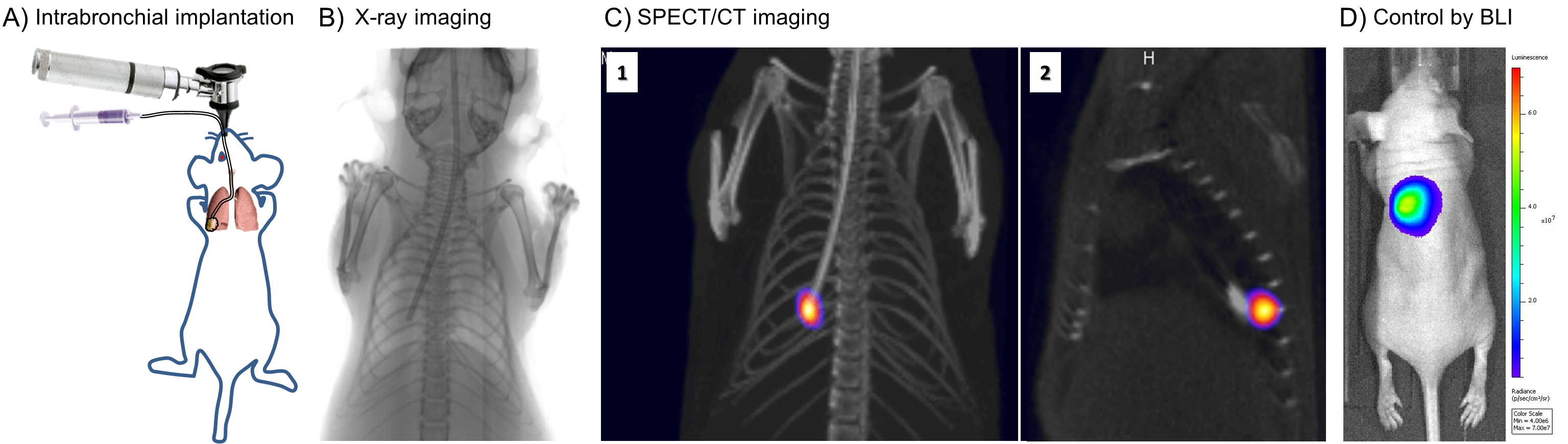

Supplement: S1 Fig — (A) The diagram represents the route of inoculation. The catheter is inserted in the deep bronchus through the trachea, so that the tumor grows in the lower lobes of the lungs, near the posterior diaphragm surface. (B) The in vivo control of the catheter positioning by planar X-ray is performed in order to avoid the implantation in the wrong site. (C1) Control of the accuracy of cell deposition into the deep bronchus by SPECT/CT imaging of 99Tcm-labeled cells. (C2) Sagittal view demonstrating the suitable location, at the posterior part of the lung. (D) Tumor growth is confirmed by BLI on day 7. (TIF) [file pone.0153532.s001.tif]

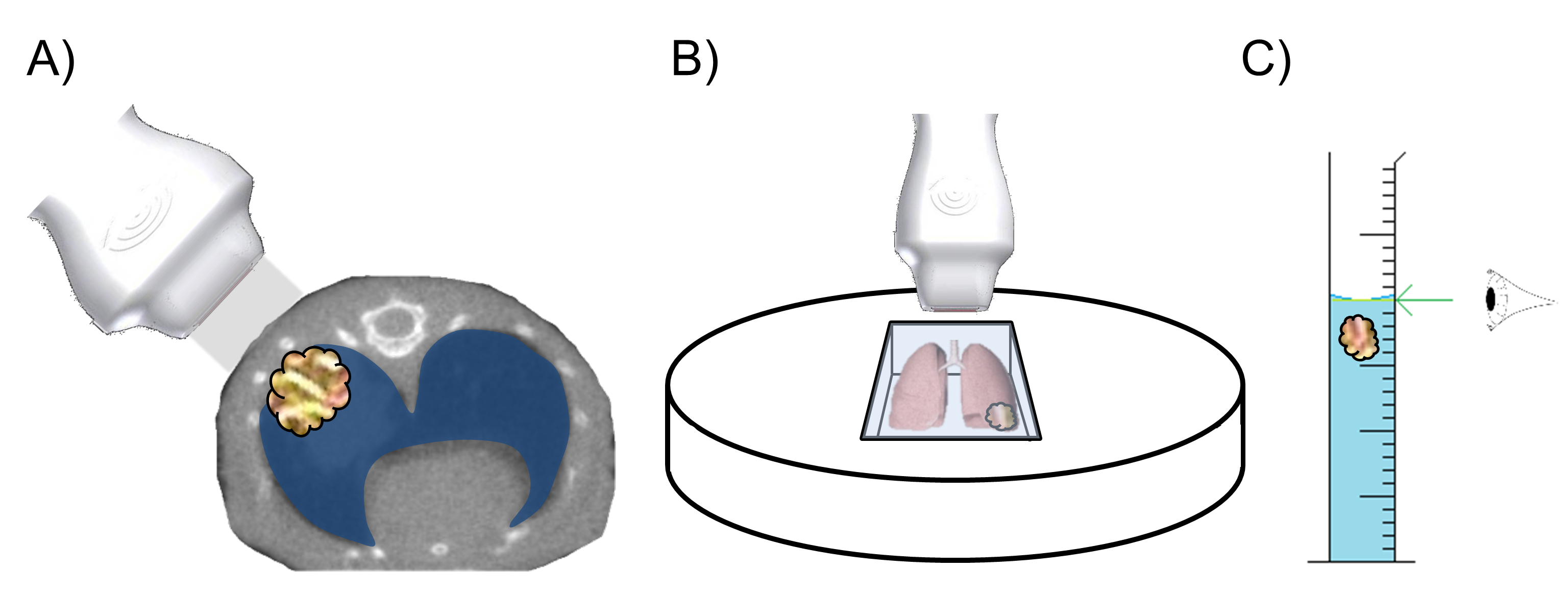

Supplement: S2 Fig — (1) Transducer positioning allowing for the conduction of ultrasound through the tumor parenchyma (delineated yellow area). (2) Set up for ex vivo 3D US acquisitions. An excavated plate is filled with ultrasound conductive gel around the tumor tissue in order to avoid any movement, and the transducer is positioned above. (3) Volumetric determination by immersing the tumor in a graduated cylinder filled with water. The water is removed from the graduated cylinder to adjust the concave meniscus at the upper edge of the baseline graduation mark and then weighed. (TIF) [file pone.0153532.s002.tif]
